# Residual Tumor Cells That Drive Disease Relapse after Chemotherapy Do Not Have Enhanced Tumor Initiating Capacity

**DOI:** 10.1371/journal.pone.0045647

**Published:** 2012-10-24

**Authors:** Ganapati V. Hegde, Cecile de la Cruz, Jeffrey Eastham-Anderson, Yanyan Zheng, E. Alejandro Sweet-Cordero, Erica L. Jackson

**Affiliations:** 1 Department of Discovery Oncology, Genentech, Inc., South San Francisco, California, United States of America; 2 Department of Pathology, Genentech, Inc., South San Francisco, California, United States of America; 3 Cancer Biology Program, Department of Pediatrics, Stanford University School of Medicine, Stanford, California, United States of America; The University of Texas M.D Anderson Cancer Center, United States of America

## Abstract

Although chemotherapy is used to treat most advanced solid tumors, recurrent disease is still the major cause of cancer-related mortality. Cancer stem cells (CSCs) have been the focus of intense research in recent years because they provide a possible explanation for disease relapse. However, the precise role of CSCs in recurrent disease remains poorly understood and surprisingly little attention has been focused on studying the cells responsible for re-initiating tumor growth within the original host after chemotherapy treatment. We utilized both xenograft and genetically engineered mouse models of non-small cell lung cancer (NSCLC) to characterize the residual tumor cells that survive chemotherapy treatment and go on to cause tumor regrowth, which we refer to as tumor re-initiating cells (TRICs). We set out to determine whether TRICs display characteristics of CSCs, and whether assays used to define CSCs also provide an accurate readout of a cell’s ability to cause tumor recurrence. We did not find consistent enrichment of CSC marker positive cells or enhanced tumor initiating potential in TRICs. However, TRICs from all models do appear to be in EMT, a state that has been linked to chemoresistance in numerous types of cancer. Thus, the standard CSC assays may not accurately reflect a cell’s ability to drive disease recurrence.

## Introduction

The identity and properties of cancer stem cells (CSCs) has been a field of intense study in recent years. CSCs have been defined as having the unique capability to both self renew and give rise to differentiated progeny in serial transplantation assays [1]. The isolation of CSCs based on distinct surface marker expression has been reported for numerous hematologic malignancies and solid tumors [2]. Several groups have reported that CSCs show enhanced resistance to conventional chemotherapeutic agents and radiation treatment [3]–[8]. Thus, it has been hypothesized that CSCs are inherently resistant to chemotherapy and as such responsible for disease relapse.

For most cancers, disease relapse after chemotherapy is a major cause of mortality. Thus, a better understanding of the cells that cause recurrence, which we call tumor re-initiating cells (TRICs), could have a major impact on our ability to effectively treat patients. This is particularly relevant for non-small cell lung cancer (NSCLC) because more than two thirds of patients are not candidates for surgical resection. Most patients present with advanced disease and are treated with chemotherapy, radiation or a combination of the two [9]. However, despite aggressive treatment the five-year survival rate for NSCLC remains at 17.5% [10]. Although CSCs have been characterized in many different cancers [11], they remain ill-defined in NSCLC [12]. Moreover, conflicting reports on the use of cell surface markers to isolate CSCs from NSCLC tumors leave their identity uncertain [13]–[17]. Finally, it is unclear how the ability of purified cell populations to initiate new tumors in a naïve host, the gold-standard CSC assay, relates to the maintenance of tumor growth or tumor relapse in a patient.

We identified several NSCLC models whose tumors regress upon treatment with standard of care chemotherapy. Despite significant cytoreduction, the residual tumors in each of these models re-grew after the cessation of therapy. As such, the residual tumor cells that survive chemotherapy treatment in these models must be the cells responsible for disease relapse and we refer to them from here on as TRICs. We isolated TRICs from each of these models and assessed them for their CSC properties using surface marker and gene expression analysis and serial transplantation assays. Our data show that TRICs do not consistently meet criteria typically used to define CSCs, but are indeed in a state of epithelial to mesenchymal transition (EMT), which has previoiusly been attributed to both stemness and drug-resistance [18], [19].

**Figure 1:**
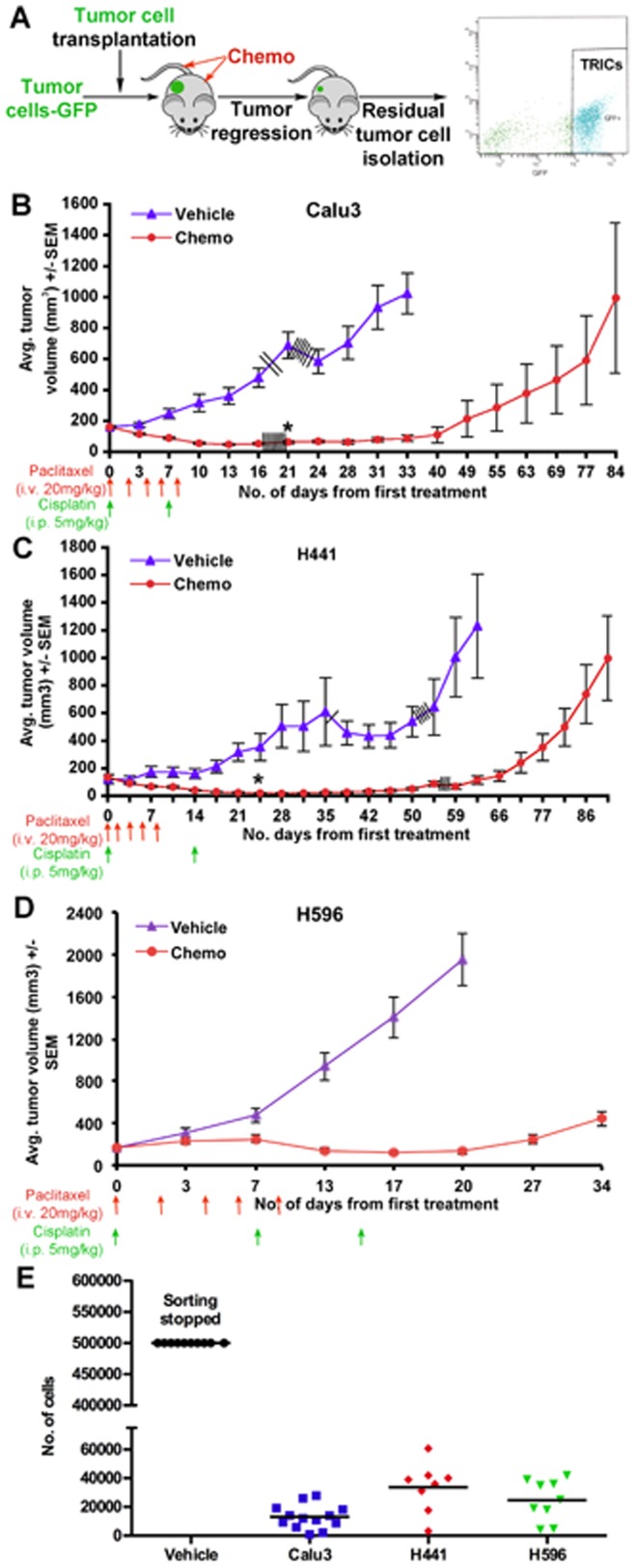
Characterization of *In vivo* Models for the Study of Tumor Re-Initiating Cells (TRICs). (A) Schematic representation of study design. GFP labeled human tumor cells were transplanted subcutaneously, and when tumor size reached ∼250 mm^3^ mice were treated with either vehicle or chemotherapy as shown in the respective models. GFP+ tumor cells were isolated from regressed or vehicle-treated tumors by FACS-sorting after enzymatic digestion and dissociation. Tumors were collected a minimum of one week after the last dose of chemotherapy and before the resumption of tumor growth. (B) Growth of vehicle and chemo-treated Calu3-GFP xenograft tumors. Data presented as mean tumor volume ± SEM, n = 15/group. (C) Growth of vehicle and chemo-treated H441-GFP xenograft tumors. Data presented as mean tumor volume ± SEM, n = 9/group for vehicle-treated and n = 14/group for chemo-treated. (D) Growth of vehicle and chemo-treated H596-GFP xenograft tumors. Data presented as mean tumor volume ± SEM, n = 10/group for vehicle-treated and n = 18/group for chemo-treated. Each/indicates a mouse euthanized for analysis. (E) Number of GFP+ tumor cells present in vehicle and chemo-treated tumors as determined by FACS. Sorting of vehicle-treated tumor cells was stopped at 500,000 cells for all models.

**Table 1:** Tumor initiating potential of TRICs in transplantation assays.

Tumor Model	No. of Cells Grafted	Treatment	No. Micewith Tumor	Tumor Volume (mm^3^)AVG ± SEM
**Calu3-GFP on day 125**	500	Vehicle (Left)	1/9	31.4±31.4
		Chemo (Right)	0/9	0
	5000	Vehicle (Left)	4/8	112.7±39.7
		Chemo (Right)	0/8	0
	25000	Vehicle (Left)	6/8	346.3±108.2
		Chemo (Right)	0/8	0
**H441-GFP on day 125**	100	Vehicle (Left)	2/5	212.6±194.5
		Chemo (Right)	0/5	0
	1000	Vehicle (Left)	3/5	261.4±149.7
		Chemo (Right)	3/5	159.4±90.1
	5000	Vehicle (Left)	5/5	1072.3±355.1
		Chemo (Right)	4/5	271.6±159.1
**H596-GFP on day 36**	500	Vehicle (Right)	4/7	78.7±25.8
		Chemo (Left)	6/7	268.3±77.2
	5000	Vehicle (Right)	6/7	262.8±60
		Chemo (Left)	6/7	883.8±450
	22000	Vehicle (Right)	5/7	210.6±75.4
		Chemo (Left)	5/7	696.5±260.8
**Tumor Model**	**No. of Cells Grafted**	**Treatment**	**No. Mice with Tumor**	**Tumor Number AVG ± SEM**
**GEMM on day 112**	100	Vehicle	0/17	0
		Chemo	3/8	0.6±0.3
	1000	Vehicle	9/19	1±0.3
		Chemo	5/10	0.8±0.1
	5000	Vehicle	17/19	3.3±0.8
		Chemo	9/9	5.8±1.4

Tumor size >50 mm^3^ was considered as positive for tumor growth.

## Materials and Methods

### Cell Culture

Calu3, H441 and H596 human NSCLC cell lines were obtained from American Type Culture Collection (ATCC), Manassas, VA. To generate GFP expressing stable cell lines, Calu3, H441 and H596 cell lines were transduced with TZV-b-actin-eGFP lentivirus. After multiple passages, the 20% highest GFP expressing cells were sorted, amplified and preserved for further studies. These sub-lines were described as Calu3-GFP, H441-GFP and H596-GFP.

**Figure 2:**
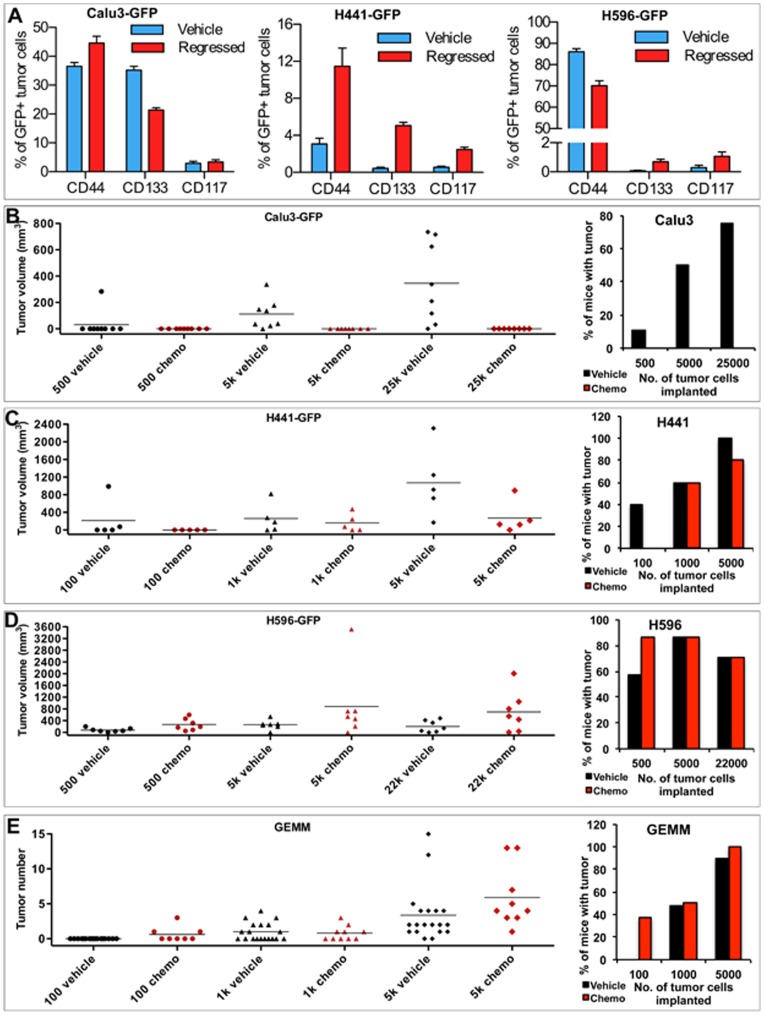
Characterization of TRICs for Cancer Stem Cell Potential *in vivo*. (A) Proportion of CSC-marker positive cells in vehicle and chemotherapy-treated Calu3, H441 and H596 tumors. Data is presented as average +/− SEM, n ≥4 mice per group. (B–D) Tumor forming capacity of B) Calu3-GFP, C) H441-GFP and D) H596-GFP tumor cells. Cells were isolated from vehicle or chemo-treated mice at tumor regression and grafted subcutaneously into athymic nude mice. Graphs show indivdual tumor size with bars indicating average tumor volume per group (left) and tumor incidence (right). Tumors >50 mm^3^ were considered as positive tumor growth. (E) Tumor forming capacity of GEMM tumor cells isolated from vehicle or chemo treated mice at tumor regression and grafted orthotopically into athymic nude mice. Graphs show number of tumors per individual mouse with bars indicating average number (left) and tumor incidence (right). (For further details see also Table 1).

**Table 2:** Effect of stroma on tumor-initiating potential of TRICs in transplantation assays.

Tumor Model	Sl. No.	Groups	Tumor Volume (mm^3^)AVG ± SEM	No. Micewith Tumor	Tumor FormationFrequency (%)
**Calu3-GFP on** **day 97**	1	5k VT + 50 k CS	642.5±113.4	9/9	100
	2	5k VT + 50 k VS	717.5±170.7	9/9	100
	3	5k VT	1509.2±312.8	9/9	100
	4	5k CT + 50 k CS	152.9±76.7	5/9	55.5
	5	5k CT + 50 k VS	257.8±89.7	6/9	66.6
	6	5k CT	251.9±130.6	4/9	44.4
	7	50k VS	103.4±55.4	3/9	33.3
	8	50k CS	0	0/9	0
	9	500 VT + 5 k CS	240.1±73.4	7/9	77.7
	10	500 VT + 5 k VS	551.9±118.8	8/9	88.8
	11	500 CT + 5 k CS	1.7±1.7	0/9	0
	12	500 CT + 5 k VS	11.3±8.3	1/9	11.1
	13	5k VS	54.4±28.9	3/9	33.3
	14	5k CS	0	0/9	0
**H441-GFP on** **day 139**	1	2.5 k VT + 25 k CS	895.3±226.6	7/8	87.5
	2	2.5 k VT + 25 k VS	958.6±243.1	7/9	77.7
	3	2.5 k CT + 25 k CS	9.8±9.8	1/14	7.1
	4	25 k VT	603.5±304.2	4/8	50
	5	25 k CS	0	0/8	0

VT = Tumor cells from vehicle-treated mice, CT = Tumor cells from chemo-treated mice at regression, VS = Stromal cells from vehicle-treated mice, and CS = Stromal cells from chemo-treated mice at regression. Tumor size of >50 mm^3^ was considered as positive for tumor growth.

#### Sphere formation assays

To determine the sphere forming potential of TRICs, tumors were dissociated and GFP+ cells were collected by FACS. Cells were resuspended in N5 media at a concentration of 40 cells/ul. The cell suspension was mixed 1∶1 with matrigel (BD Biosciences) and 100 ul/well of the cell/matrigel solution was plated into 96 well plates. Plates were incubated at 37°C for 30–60 minutes to allow solidification of the matrigel, then overlayed with 100 ul of N5 media. Cells were cultured for 7 days at 37°C then assessed for sphere formation. N5 media consisted of DMEM/F12 (+HEPES/glutamine), 5% FBS, bovine pituitary extract (35 ug/ml), N2 supplement, antibiotic/antimitotic, EGF (20 ng/ml) and FGF (20 ng/ml).

**Figure 3:**
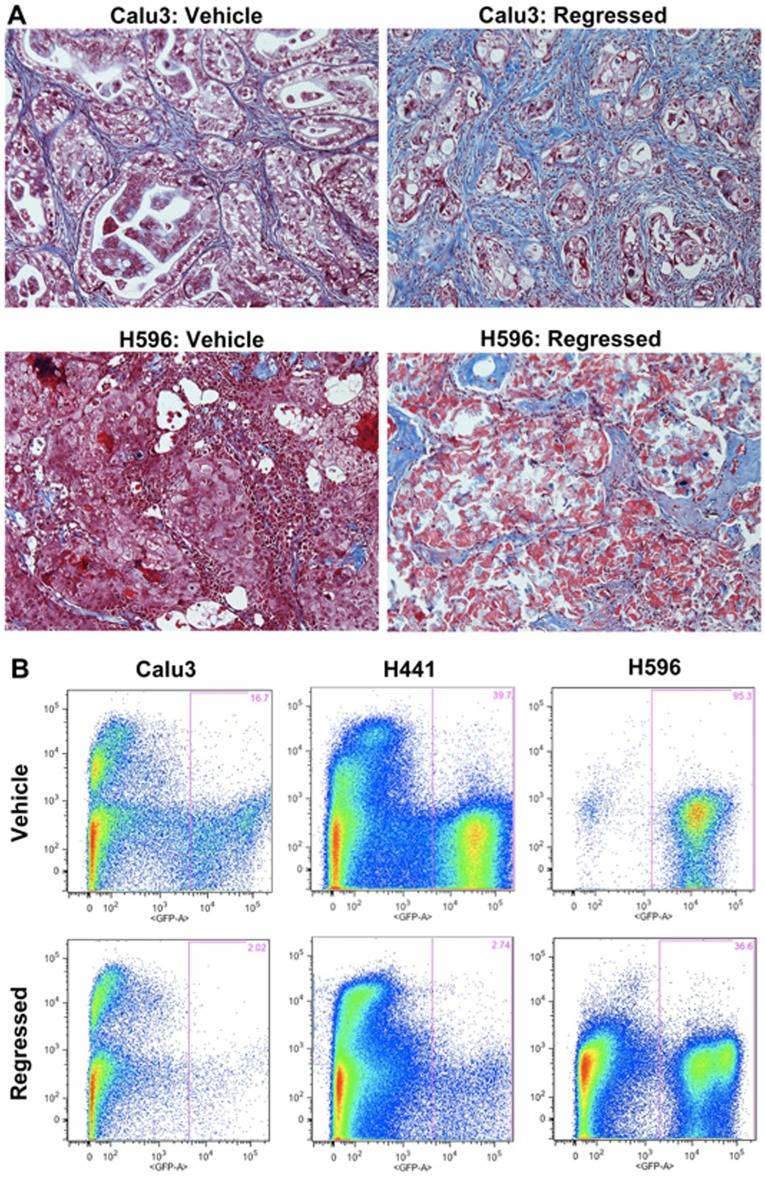
Increased Stromal Component in Residual Chemotherapy-Treated Tumors. A) Masson’s Trichrome staining of vehicle and regressed chemo-treated tumors showing a decrease in tumor cells (red) and an increase in collagen-containing stromal cells (blue) in residual tumors. B) FACS analysis of vehicle and regressed tumors showing a decreased tumor:stroma ratio in residual tumors.

### Generation of Donor Xenograft Tumors

All animal experiments were approved by the Institutional Animal Care and Use Committee (IACUC) at Genentech Inc. Athymic nude mice were housed and maintained in pathogen-free conditions. To generate tumors, suspensions of freshly passaged tumor cells (15–20 million) were transplanted subcutaneously into the right flank of athymic nude mice. When tumors reached ∼150–250 mm^3^, the mice were divided into different treatment groups. Mice were then treated with either vehicle or chemotherapy (paclitaxel, i.v. + cisplatin, i.p.). The chemotherapy-dosing regimen was paclitaxel 20 mg/kg i.v. every other day for 5 doses, and cisplatin 5 mg/kg i.p. on days 1 and 7 for the Calu3 model, days 1 and 14 for the H441 model, and days 1, 7 and 14 for the H596 model. Regressed tumors from chemo-treated and time matched tumors from vehicle-treated control mice were collected at least 1 week after the last dose of chemotherapy. Tumors were minced and dissociated using dispase/collagenase. Propidium iodide (PI) was used to exclude dead cells and GFP+ tumor cells were sorted using FACSVantage and FACSAria (BD Biosciences).

### Flow Cytometric Analysis

GFP+ tumor cells isolated from vehicle and chemo-treated mice at regression were stained with CD133-APC (MACS #130-090-826), CD44-PE (eBioscience #12-0441-83) and CD117-PEC-Cy7 (BD Bioscience # 339195) antibodies or appropriate isotype-matched control antibodies. Samples were analyzed using the FACScaliber and data was analyzed using FlowJo software.

**Figure 4:**
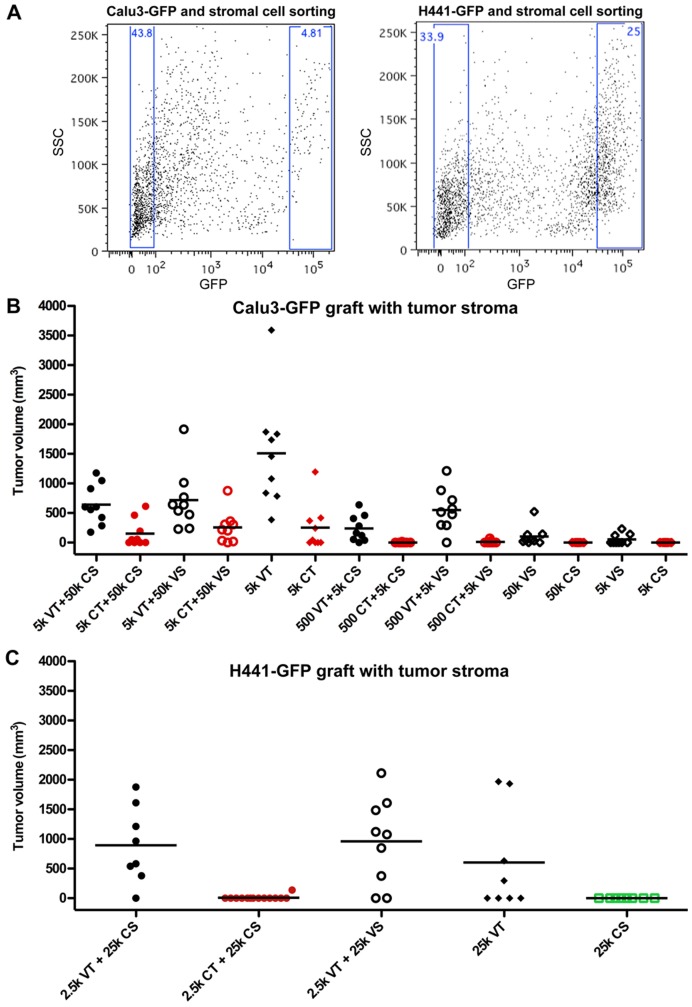
Effect of Stroma on Tumor Initiating Potential of TRICs in transplanation assays. A) Representative FACS plots showing gates used for sorting GFP+ tumor and stromal cells from Calu3 (left) and H441 tumors (right). B) Calu-3 and C) H441 tumor (GFP+) and stromal (GFP-) cells were isolated from tumors taken from vehicle or chemo-treated mice at tumor regression. Tumor and stromal cells were mixed as indicated and grafted subcutaneously into athymic nude mice. Graphs show individual tumor volumes at the end of study, bars indicate the average tumor volume per group. See Table 2 for further details.

### RNA Isolation, cDNA Preparation and qRT-PCR

RNA was isolated from FACS sorted tumor cells isolated from vehicle-treated and chemo-treated mice at regression using Qiagen RNeasy Micro Kit. Complementary DNA was prepared from total RNA using ABI High capacity cDNA reverse transcription kit according to manufacturer’s instructions. Expression of *VIMENTIN*, *ZEB1*, *ZEB2*, *SNAI1*, *SNAI2*, *TWIST1* and *N-CADHERIN* was determined using ABI gene specific primers/probe by quantitative real time PCR (ABI 7500). Gene expression was normalized using GAPDH house keeping gene.

### Serial Transplantation of Xenograft Tumor Cells to Assess Tumor Initiating Potential

Propidium iodide (PI)^-^, GFP+ tumor cells were sorted by FACS from tumors of vehicle or chemotherapy-treated mice at regression in Calu3, H441 and H596 models. Cells were counted using trypan blue staining to exclude dead cells and debris and cell suspensions were prepared in 1∶1 mixture of RPMI and matrigel (BD Bioscience). These cell suspensions were injected subcutaneously into athymic nude mice, with vehicle-treated and chemo-treated tumor cells being injected into opposite flanks. Tumor growth was monitored for the amount of time indicated.

**Figure 5:**
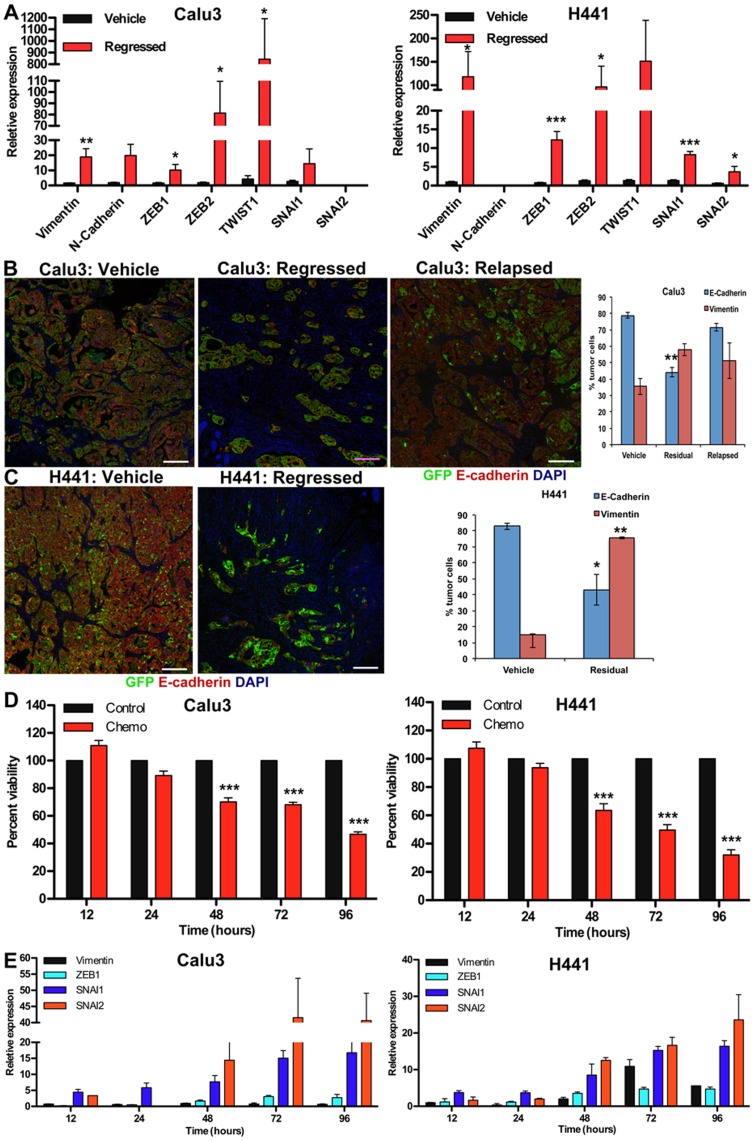
TRICs are in a state of EMT. (A) Expression of EMT markers and EMT-driving transcription factors in Calu3-GFP and H441-GFP tumor cells isolated from vehicle or chemo treated mice at tumor regression was assessed by qRT-PCR. (B–C) Proportions of E-Cadherin and Vimentin expressing tumor cells were determined by immunofluorescence analysis in B) Calu3-GFP and C) H441-GFP tumors. Sections were co-stained for GFP and E-Cadherin or GFP and Vimentin. Data are expressed as average+/−SEM. (D–E) Correlation between the time course of chemotherapy-induced cell death (D) and expression of EMT-drivers as determined by qRT-PCR (E) Presented expression levels are relative to time-matched vehicle-treated controls.

### Orthotopic Implantation of GEMM Tumor Cells to Assess Tumor Initiating Potential

Beginning 12 weeks after tumor initiation, LSL-*Kras^G12D^;p53^Fl/Fl^* mice were treated with cisplatin (7 mg/kg IP) or vehicle once a week for 3 weeks. Lungs were collected from cisplatin and vehicle-treated mice one week after the final dose of cisplatin. Lungs were dissociated and GFP+ tumor cells were collected by FACS. Cells were counted using trypan blue staining to exclude dead cells and debris, and equal numbers of vehicle- and chemo-treated tumor cells were implanted orthotopically via intratracheal intubation. Lungs were collected 16 weeks later and tumor burden was assessed by *ex vivo* micro-CT.

### Micro-CT Analysis of Lungs

Mice were euthanized by transcardiac perfusion of PBS under anesthesia. A cannula was inserted through the trachea. Intact tumor bearing lungs were dissected, fixed with 10% formalin for 24 hours and further processed as described previously [20]. Lungs were imaged ex-vivo with a MicroCT 42 (SCANCO Medical, Basserdorf, Switzerland) x-ray micro-computed tomography (micro-CT) system. Images were generated by operating the x-ray tube at an energy level of 45 kV, a current of 177 µA and an integration time of 300 milliseconds.

### Influence of Stroma on Tumor-initiating Potential

To assess the effect of stroma on tumor initiating potential, four different cell fractions were isolated from tumors from vehicle- or chemo-treated mice by FACS sorting tumors from several mice. The following cell types from were pooled from multiple tumors (1) GFP+ tumor cells from chemo-treated mice (CT), (2) GFP- stromal cells from chemo-treated mice (CS), (3) GFP+ tumor cells from vehicle-treated mice (VT), and (4) GFP- stromal cells from vehicle-treated mice (VS). Tumor cells and stromal cells were mixed 1∶10 in the combinations indicated, and transplanted subcutaneously into the left and right flanks of athymic nude mice. Tumor growth was monitored for the duration indicated.

### Immunoflurescence Analysis

For histological evaluation of tumors, tumor tissues were collected from vehicle- or chemo-treated mice at regression and relapse and were fixed in formalin. Tissues were paraffin embedded and cut into 3 uM sections. Immunofluorescence was performed following Declere dewaxing/unmasking according to manufacturer’s instructions (Sigma-Aldrich D3565). Chicken-Anti-GFP (Aves Labs cat# GFP-1020) was used at a 1∶500 dilution, Rabbit-anti-E-Cadherin (Cell Signaling cat# 3195) at a 1∶75 dilution and Rabbit-anti-Vimentin (clone SP20 Lab Vision cat# RB-9120-S1) at a 1∶200 dilution. Secondary antibodies were Alexa-488 Goat-anti-Chicken (Invitrogen) at a 1∶500 dilution and Alexa-594 Donkey-anti-Rabbit (Invitrogen) 1∶800. Nuclei were identified by DAPI.

### Statistical Analysis

Results are presented as average +/− SEM, and statistical significance was determined with an unpaired *t* test. p<0.05 was considered as statistically significance.

## Results

### *In vivo* Models of NSCLC for the Study of Residual Disease and Relapse

To characterize a subpopulation of tumor cells that survive chemotherapy treatment and mediate tumor recurrence, we identified several *in vivo* xenograft models that show tumor regression or stasis followed by relapse in response to standard-of-care chemotherapeutic agents (Fig. 1A). We used GFP-expressing sublines of the Calu3, H441 and H596 human NSCLC xenograft models. Established tumor bearing mice were treated with either vehicle or the maximum tolerated dose of a combination regimen of paclitaxel and cisplatin. Following completion of treatment, tumor volumes in chemo-treated mice were similar to (H596) or smaller than (Calu3, H441) the volume at study start (Fig. 1B–D). Regression persisted for several weeks after the last dose of chemotherapy but all tumors subsequently recurred (Fig. 1B–D). In addition, we used the *LSL-K-ras^G12D^;p53^Fl/Fl^* genetically engineered mouse model (GEMM) of NSCLC [21] crossed to a RFP Cre-reporter strain [22]. Cisplatin treatment of *LSL-K-ras^G12D^;p53^Fl/Fl^* mice prolongs survival but mice still rapidly succumb to their disease indicating that tumors initially respond to therapy but resume growing after treatment [23].

Although each of the models used responded to chemotherapy, the tumors relapsed at varying times after therapy even when there was nearly complete cytoreduction. We next sought to isolate the GFP- or RFP-labeled tumor cells that survived after chemotherapy but prior to the onset of tumor re-growth, since these cells by definition are enriched for the TRIC population. In each of the 3 xenograft models the number and proportion of GFP+ tumor cells present in chemo-treated animals was significantly lower than in vehicle-treated control mice (Fig. 1E) indicating that only a small proportion of tumor cells survive after chemotherapy.

### Characterization of TRICs for Cancer Stem Cell Phenotype

To determine the expression of known CSCs markers in TRICs, we carried out *in vivo* studies as described above and used flow cytometry to analyze the proportion of GFP+ tumor cells in residual and vehicle-treated tumors that express the previously reported NSCLC CSC markers, CD133, CD44 and CD117. The proportion of CD133+ and CD44+ tumor cells varied greatly between models (Fig. 2A), consistent with the wide variation in the proportion of positive cells between tumors noted in the original publications identifying each of these CSC markers [24], [25]. In the H441 model (Fig. 2A), there was a robust increase in CSC marker positive tumor cells with a ∼3-fold increase in CD44+ cells, a 12.5-fold increase in CD133+ cells and a 5-fold increase in CD117+ cells (Fig. 2A). In contrast, in the Calu3 model there was a modest but significant increase in the proportion of CD44+ tumor cells, a significant decrease in the proportion of CD133+ tumor cells and no change in the proportion of CD117+ cells in tumors from chemo-treated mice. Results were also mixed in the H596 model (Fig. 2A), with a significant decrease in the proportion of CD44+ cells, an increase in CD133+ cells and no significant change in CD117+ tumor cells. Thus, using previously described CSC markers we did not observe the consistent enrichment of a specific tumor cell population.

To further explore whether the cells mediating tumor relapse are indeed CSCs, we assessed the tumor-initiating capacity of TRICs. We were unable to determine the tumorigenicity of these cells using sphere assays, since cells from vehicle and chemo-treated Calu3, H441 or H596 tumors failed to generate spheres after being grown *in vivo*, despite robust sphere formation from the parental and GFP-stable cell lines under identical conditions.

Next, we conducted transplantation assays to assess the tumor-initiating capacity of TRICs isolated from the Calu3, H441 and H596 xenograft models and the *LSL-K-ras^G12D^*;*p53^Fl/Fl^* GEMM. Surprisingly, for both the Calu3 and H441 models, tumor cells isolated from vehicle-treated mice were more efficient at tumor initiation than tumor cells from chemo-treated mice (Fig. 2B–C, Table 1). Although the incidence of tumor formation from H441 vehicle- and chemo-treated cells was similar, the growth of the vehicle-treated tumor cells was considerably more robust as demonstrated by the significantly larger average tumor volume. In contrast, tumor cells isolated from chemo-treated H596 tumors were significantly more tumorigenic than cells isolated from vehicle-treated tumors (Fig. 2D, Table 1). Here again, tumor incidence was similar between vehicle- and chemo-treated tumor cells, but tumors derived from vehicle-treated tumor cells were significantly larger. There was no significant difference in the tumor initiating potential of *LSL-K-ras^G12D^*;*p53^Fl/Fl^* GEMM tumor cells isolated from chemo- or vehicle-treated mice upon orthotopic grafting via intratracheal intubation (Fig. 2E, Table 1). Thus, TRICs are not consistently enriched for CSCs. Furthermore, the results of the transplantation studies showed no correlation between tumor initiating capacity and the expression of CSCs markers. TRICs isolated from the H441 model were enriched for all CSCs markers (Fig. 2A) but did not show increased tumor initiating potential, while TRICs isolated from the H596 model demonstrated increased tumor initiating potential but were only enriched for CD133 expression (Fig. 2A).

Histological examination and FACS analysis of vehicle-treated and regressed tumors revealed dramatic differences in the amount of stroma between models, and between vehicle and regressed tumors within a given model (Fig. 3A–B). Furthermore, the size of the stromal component in the residual tumors was inversely correlated with tumor initiating potential of the TRICs. Of the 3 models, residual H596 tumors contained the least stroma and H596 TRICs were significantly more tumorigenic than cells from vehicle-treated mice. In contrast, residual Calu3 and H441 tumors were comprised mainly of stroma and TRICs isolated from these tumors were limited in their tumor initiating capacity upon transplantation. We reasoned that lack of stromal support could explain why TRICs readily re-grew tumors in the original host but showed decreased tumorigenicity upon transplantation. To determine if TRIC growth was dependent on stromal-derived factors, we performed transplantation studies in the presence of stromal cells. We isolated four different cell fractions from tumors from chemotherapy or vehicle-treated mice by FACS sorting (Fig. 4A): (1) GFP+ tumor cells from chemo-treated mice at regression (CT), (2) GFP- stromal cells from chemo-treated mice at regression (CS), (3) GFP+ tumor cells from vehicle-treated mice (VT), and (4) GFP- stromal cells from vehicle-treated mice (VS). We mixed tumor and stromal cells and transplanted the mixtures into naïve host mice. In the Calu3 model neither VS nor CS cells provided a growth benefit to VT or CT cells, and as observed earlier, VT cells were more tumorigenic than CT cells when implanted alone (Fig. 4B, Table 2). There was a significant growth advantage for VT cells from the H441 model when they were implanted with either VS or CS but CT cells were still much less tumorigenic than VT cells even when mixed with stroma (Fig. 4C, Table 2). Of note, the tumor-initiating potential of the TRICs in the transplantation assay was not fully consistent between experiments. Transplantation of 5,000 Calu3 TRICs did not result in tumor formation in any of the grafted animals in the previous study (Fig. 2B and Table 1) but generated tumors in 44% of recipients in this study (Fig. 4B, Table 2). The results of H441 TRIC transplants were also inconsistent, with TRICs generating tumors in the previous study but not in this one (Fig. 2C, Table 1 and Fig. 4C, Table 2 respectively). These results suggest that small variations in experimental conditions could dramatically affect tumor-initiating potential upon transplantation. However, in both experiments Calu3 and H441 TRICs were less tumorigenic than tumor cells from vehicle-treated mice, demonstrating that TRICs are not consistently enriched for CSCs as evidenced by standard transplantation assay.

### Characterization of TRICs for EMT Phenotype

Emerging evidence suggests that tumor cells undergoing EMT have an increased capacity for chemoresistance, metastasis and tumor relapse [26]–[28]. EMT has also been shown to confer cancer stem cell properties in breast, colon and pancreatic cancers [18], [29]. Therefore, we studied TRICs for EMT characteristics. We assessed the expression of markers and regulators of EMT in TRICs and vehicle-treated tumor cells by qRT-PCR. There was a significant increase in the expression of mesenchymal markers and EMT-inducing transcription factors in residual tumor cells (Fig. 5A). In addition, we also collected tumor specimens for histological analysis at regression and relapse. Immunofluorescence analysis of these specimens corroborated the qRT-PCR results. There was a significant increase in the proportion of Vimentin expressing tumor cells and significant decrease in the proportion of E-Cadherin expressing tumor cells in regressed tumors compared to tumors from vehicle-treated mice (Fig. 5B–C). The proportions in relapsed tumors were similar to the vehicle-treated tumors demonstrating the plasticity of the TRICs. Together, these results demonstrate that TRICs are in an EMT state.

To further assess whether chemotherapy treatment induces the expression of EMT-inducing transcription factors or selects for a population of cells that are in an EMT state, we conducted a time course analysis. We found that the onset of enrichment of EMT-inducing transcription fractors in cells treated with chemotherapy in vitro coincides with the onset of cell death, and shows additional enrichment as additional cells are lost from the cultures (Fig. 5D–E). These data suggest that chemotherapy is indeed selecting for a population of cells in EMT.

## Discussion

In the cancer stem cell model of tumorigenesis, a small subset of cancer cells has the unique capacity to propagate the tumor due to their exclusive ability to self-renew and to generate progeny that differentiate into the heterogenous non-tumorigenic cell types that make up the bulk of the tumor [1]. Furthermore, several studies have shown that CSCs have increased resistance to conventional chemo- and radio-therapies [6], [30], [31]. The CSC hypothesis has received key attention because it provides a possible explanation for the processes of disease relapse after therapy [32]. However, because CSCs have been defined by transplantation assays, the roles of CSCs in maintaining an established tumor and in disease relapse within the original host remain to be determined. In this notion, we utilized several *in vivo* preclinical models treated with standard of care chemotherapy agents [33] to characterize the cells responsible for tumor relapse, TRICs. By utilizing models that regress in response to chemotherapy and models that undergo stasis, we aimed to reflect the different responses of NSCLC patients to chemotherapy. We isolated and evaluated them for several properties commonly used to define CSCs.

Numerous publications regarding the use of self-surface markers to identify NSCLC CSCs report conflicting results. For example, CD133+ cells from NSCLC cell lines and primary patient samples have been reported to have the unique ability to generate spheres in vitro and to initiate tumors in immuno-compromised mice [12], [13], [16], [17], [34]. In contrast, others report that both the CD133+ and CD133- cells display similar colony formation, self-renewal, proliferation, differentiation, invasion, chemoresistance and tumorigenicity [14], [16]. Furthermore, CD133 expression showed no correlation with disease-free or overall survival of NSCLC patients. [17]. The use of CD44 to identify NSCLC CSCs has generated similar conflicting results [15]. We, too, saw inconsistent enrichment of these putative CSC markers in residual tumor cells from multiple models. Moreover, even in models where the proportion of CD133+ or CD44+ cells is enriched, the TRICs do not have an enhanced tumor initiating capacity.

Because the use of cell surface markers for the prospective identification of CSCs from NSCLCs has yielded conflicting results, we also compared the tumor initiating potential of TRICs vs. vehicle-treated tumor cells from xenograft and GEM models. However, TRICs from only one xenograft model were enriched for tumor initiating potential. It is important to note that when left undisturbed within the original host TRICs consistently caused tumor recurrence after chemotherapy in all of the models we studied, even in rare instances when no palpable tumor was present after treatment. Consistent with our findings, Yan and colleagues recently reported that *in vitro*-derived, drug-tolerant cancer cell lines are less proliferative than their parental cell lines and have reduced cloning efficiency and tumor-initiating capacity [35].

We found that the majority of cells present in the residual tumors were not cancer cells, but rather stromal cells. Recent work by Gilbert and Hemann demonstrated that chemotherapy induces the release of paracrine factors from tumor-associated stromal cells modulating tumor cell survival [36]. We reasoned that the lack of necessary stromal support could explain why the transplantation of TRICs to a new host significantly decrease their tumorigenicity. Therefore, we assessed whether vehicle and chemo-treated stromal cells (VS and CS respectively) could enhance tumor initiating potential in mixing experiments. We did find that stromal cells enhanced the tumor initiating potential of both vehicle and chemo-treated H441 cells. However, this property of the stromal cells was independent of chemotherapy treatment since both VS and CS cells augmented tumor growth to a similar degree. Moreover, H441 TRICs were still less tumorigenic than cells isolated from vehicle-treated tumors, and TRICs from Calu3 tumors still had very little tumor initiating capacity even when mixed with stroma. This suggests that maintenance of the complex cell-cell and cell-extracellular matrix interactions present in regressed tumors is required for tumor regrowth to occur. Thus, our data caution about relying on classic CSC transplantation assays as a read out of disease relapse.

The EMT state has been linked to resistance to both conventional and targeted therapeutics in a variety of cancer cell lines [37]–[41]. We also found that TRICs are in an EMT state as evidenced by decreased expression of E-cadherin and increased expression of mesenchymal markers and EMT-inducing transcription factors. Thus, the EMT state of the TRICs likely contributes to their ability to withstand chemotherapy treatment. Interestingly, Mani *et al.* recently reported that induction of EMT in transformed mammary epithelial cells converts differentiated tumor cells into CSCs [18], and induction of EMT by TGF-beta was also shown to increase the stemness characteristic of NSCLC cells *in vitro* [27], [42], [43]. However, despite the EMT state of the TRICs, we did not find evidence of increased stemness, indicating that EMT may contribute to chemoresistance in a manner that is independent of its ability to confer stemness.

In conclusion, we show here that residual tumor cells that survive chemotherapy and cause disease relapse are in an EMT state but do not consistently demonstrate increased CSC marker expression or tumor initiating capacity. Our results indicate that while the analysis of known CSC markers, and the use of classical transplantation assays clearly identify tumor cells with unique and important characteristics, they may not truly identify the subset of tumor cells responsible for recurrence after chemotherapy. Rather these cells must be identified based on their abilities to withstand chemotherapy and re-initiate tumor growth. Further analysis of the cells that have been functionally defined as TRICs will likely yield novel insights into the drivers of chemoresistance and disease recurrence.

## References

[1] Clarke MF, Dick JE, Dirks PB, Eaves CJ, Jamieson CH, et al (2006) Cancer stem cells–perspectives on current status and future directions: AACR Workshop on cancer stem cells. Cancer Res 66: 9339–9344. 16990346 10.1158/0008-5472.CAN-06-3126

[2] Frank NY, Schatton T, Frank MH (2010) The therapeutic promise of the cancer stem cell concept. J Clin Invest 120: 41–50. 20051635 10.1172/JCI41004 PMC2798700

[3] Costello RT, Mallet F, Gaugler B, Sainty D, Arnoulet C, et al (2000) Human acute myeloid leukemia CD34+/CD38− progenitor cells have decreased sensitivity to chemotherapy and Fas-induced apoptosis, reduced immunogenicity, and impaired dendritic cell transformation capacities. Cancer Res 60: 4403–4411. 10969785

[4] Matsui W, Huff CA, Wang Q, Malehorn MT, Barber J, et al (2004) Characterization of clonogenic multiple myeloma cells. Blood 103: 2332–2336. 14630803 10.1182/blood-2003-09-3064 PMC3311914

[5] Dean M, Fojo T, Bates S (2005) Tumour stem cells and drug resistance. Nat Rev Cancer 5: 275–284. 15803154 10.1038/nrc1590

[6] Bao S, Wu Q, McLendon RE, Hao Y, Shi Q, et al (2006) Glioma stem cells promote radioresistance by preferential activation of the DNA damage response. Nature 444: 756–760. 17051156 10.1038/nature05236

[7] Phillips TM, McBride WH, Pajonk F (2006) The response of CD24(−/low)/CD44+ breast cancer-initiating cells to radiation. J Natl Cancer Inst 98: 1777–1785. 17179479 10.1093/jnci/djj495

[8] Dylla SJ, Beviglia L, Park IK, Chartier C, Raval J, et al (2008) Colorectal cancer stem cells are enriched in xenogeneic tumors following chemotherapy. PLoS One 3: e2428. 18560594 10.1371/journal.pone.0002428 PMC2413402

[9] (2005) Lung Cancer Principles and Practice; Harvey I. Pass DPC, JDavid H. Johnson, John D. Minna, Andrew T. Turrisis, III, editor. Philadelphia: Lippincott Williams & Wilkins.

[10] Howlader N NA, Krapcho M, Neyman N, Aminou R, Altekruse SF, Kosary CL, Ruhl J, Tatalovich Z, Cho H, Mariotto A, Eisner MP, Lewis DR, Chen HS, Feuer EJ, Cronin KA (eds). SEER Cancer Statistics Review, 1975–2009 (Vintage 2009 Populations), National Cancer Institute. Bethesda, MD. http://seer.cancer.gov/csr/1975_2009_pops09/, (based on November 2011 SEER data submission, posted to the SEER web site, April 2012. Accessed September 19, 2012).

[11] Pietras A (2011) Cancer stem cells in tumor heterogeneity. Adv Cancer Res 112: 255–281. 21925307 10.1016/B978-0-12-387688-1.00009-0

[12] Wiwanitkit V (2010) CD133 and non-small-cell lung cancer. Eur J Cardiothorac Surg 37: 988; author reply 988–989. 10.1016/j.ejcts.2009.10.027 19945887

[13] Eramo A, Lotti F, Sette G, Pilozzi E, Biffoni M, et al (2008) Identification and expansion of the tumorigenic lung cancer stem cell population. Cell Death Differ 15: 504–514. 18049477 10.1038/sj.cdd.4402283

[14] Cui F, Wang J, Chen D, Chen YJ (2011) CD133 is a temporary marker of cancer stem cells in small cell lung cancer, but not in non-small cell lung cancer. Oncol Rep 25: 701–708. 21174061 10.3892/or.2010.1115

[15] Leung EL, Fiscus RR, Tung JW, Tin VP, Cheng LC, et al (2010) Non-small cell lung cancer cells expressing CD44 are enriched for stem cell-like properties. PLoS One 5: e14062. 21124918 10.1371/journal.pone.0014062 PMC2988826

[16] Meng X, Li M, Wang X, Wang Y, Ma D (2009) Both CD133+ and CD133− subpopulations of A549 and H446 cells contain cancer-initiating cells. Cancer Sci 100: 1040–1046. 19385971 10.1111/j.1349-7006.2009.01144.x PMC11159162

[17] Janikova M, Skarda J, Dziechciarkova M, Radova L, Chmelova J, et al (2010) Identification of CD133+/nestin+ putative cancer stem cells in non-small cell lung cancer. Biomed Pap Med Fac Univ Palacky Olomouc Czech Repub 154: 321–326. 21293543 10.5507/bp.2010.048

[18] Mani SA, Guo W, Liao MJ, Eaton EN, Ayyanan A, et al (2008) The epithelial-mesenchymal transition generates cells with properties of stem cells. Cell 133: 704–715. 18485877 10.1016/j.cell.2008.03.027 PMC2728032

[19] Iwatsuki M, Mimori K, Yokobori T, Ishi H, Beppu T, et al (2010) Epithelial-mesenchymal transition in cancer development and its clinical significance. Cancer Sci 101: 293–299. 19961486 10.1111/j.1349-7006.2009.01419.x PMC11159985

[20] Caunt M, Mak J, Liang WC, Stawicki S, Pan Q, et al (2008) Blocking neuropilin-2 function inhibits tumor cell metastasis. Cancer Cell 13: 331–342. 18394556 10.1016/j.ccr.2008.01.029

[21] Jackson EL, Willis N, Mercer K, Bronson RT, Crowley D, et al (2001) Analysis of lung tumor initiation and progression using conditional expression of oncogenic K-ras. Genes Dev 15: 3243–3248. 11751630 10.1101/gad.943001 PMC312845

[22] Luche H, Weber O, Nageswara Rao T, Blum C, Fehling HJ (2007) Faithful activation of an extra-bright red fluorescent protein in “knock-in” Cre-reporter mice ideally suited for lineage tracing studies. Eur J Immunol 37: 43–53. 17171761 10.1002/eji.200636745

[23] Oliver TG, Mercer KL, Sayles LC, Burke JR, Mendus D, et al (2010) Chronic cisplatin treatment promotes enhanced damage repair and tumor progression in a mouse model of lung cancer. Genes Dev 24: 837–852. 20395368 10.1101/gad.1897010 PMC2854397

[24] Singh SK, Clarke ID, Terasaki M, Bonn VE, Hawkins C, et al (2003) Identification of a cancer stem cell in human brain tumors. Cancer Res 63: 5821–5828. 14522905

[25] Al-Hajj M, Wicha MS, Benito-Hernandez A, Morrison SJ, Clarke MF (2003) Prospective identification of tumorigenic breast cancer cells. Proc Natl Acad Sci U S A 100: 3983–3988. 12629218 10.1073/pnas.0530291100 PMC153034

[26] Wang Z, Li Y, Ahmad A, Banerjee S, Azmi AS, et al (2011) Pancreatic cancer: understanding and overcoming chemoresistance. Nat Rev Gastroenterol Hepatol 8: 27–33. 21102532 10.1038/nrgastro.2010.188

[27] Zhang HJ, Wang HY, Zhang HT, Su JM, Zhu J, et al (2011) Transforming growth factor-beta1 promotes lung adenocarcinoma invasion and metastasis by epithelial-to-mesenchymal transition. Mol Cell Biochem 355: 309–314. 21695462 10.1007/s11010-011-0869-3

[28] Gupta PB, Onder TT, Jiang G, Tao K, Kuperwasser C, et al (2009) Identification of selective inhibitors of cancer stem cells by high-throughput screening. Cell 138: 645–659. 19682730 10.1016/j.cell.2009.06.034 PMC4892125

[29] Wellner U, Schubert J, Burk UC, Schmalhofer O, Zhu F, et al (2009) The EMT-activator ZEB1 promotes tumorigenicity by repressing stemness-inhibiting microRNAs. Nat Cell Biol 11: 1487–1495. 19935649 10.1038/ncb1998

[30] Li S, Li D (2007) Stem cell and kinase activity-independent pathway in resistance of leukaemia to BCR-ABL kinase inhibitors. J Cell Mol Med 11: 1251–1262. 18205699 10.1111/j.1582-4934.2007.00108.x PMC4401291

[31] Diehn M, Cho RW, Lobo NA, Kalisky T, Dorie MJ, et al (2009) Association of reactive oxygen species levels and radioresistance in cancer stem cells. Nature 458: 780–783. 19194462 10.1038/nature07733 PMC2778612

[32] Reya T, Morrison SJ, Clarke MF, Weissman IL (2001) Stem cells, cancer, and cancer stem cells. Nature 414: 105–111. 11689955 10.1038/35102167

[33] Schiller JH, Harrington D, Belani CP, Langer C, Sandler A, et al (2002) Comparison of four chemotherapy regimens for advanced non-small-cell lung cancer. N Engl J Med 346: 92–98. 11784875 10.1056/NEJMoa011954

[34] Tirino V, Camerlingo R, Franco R, Malanga D, La Rocca A, et al (2009) The role of CD133 in the identification and characterisation of tumour-initiating cells in non-small-cell lung cancer. Eur J Cardiothorac Surg 36: 446–453. 19464919 10.1016/j.ejcts.2009.03.063

[35] Yan H, Chen X, Zhang Q, Qin J, Li H, et al (2011) Drug-tolerant cancer cells show reduced tumor-initiating capacity: depletion of CD44 cells and evidence for epigenetic mechanisms. PLoS One 6: e24397. 21935404 10.1371/journal.pone.0024397 PMC3174165

[36] Gilbert LA, Hemann MT (2010) DNA damage-mediated induction of a chemoresistant niche. Cell 143: 355–366. 21029859 10.1016/j.cell.2010.09.043 PMC2972353

[37] Wang D, Shen Q, Chen YQ, Wang MH (2004) Collaborative activities of macrophage-stimulating protein and transforming growth factor-beta1 in induction of epithelial to mesenchymal transition: roles of the RON receptor tyrosine kinase. Oncogene 23: 1668–1680. 15001985 10.1038/sj.onc.1207282

[38] Yang AD, Fan F, Camp ER, van Buren G, Liu W, et al (2006) Chronic oxaliplatin resistance induces epithelial-to-mesenchymal transition in colorectal cancer cell lines. Clin Cancer Res 12: 4147–4153. 16857785 10.1158/1078-0432.CCR-06-0038

[39] Thomson S, Buck E, Petti F, Griffin G, Brown E, et al (2005) Epithelial to mesenchymal transition is a determinant of sensitivity of non-small-cell lung carcinoma cell lines and xenografts to epidermal growth factor receptor inhibition. Cancer Res 65: 9455–9462. 16230409 10.1158/0008-5472.CAN-05-1058

[40] Kajiyama H, Shibata K, Terauchi M, Yamashita M, Ino K, et al (2007) Chemoresistance to paclitaxel induces epithelial-mesenchymal transition and enhances metastatic potential for epithelial ovarian carcinoma cells. Int J Oncol 31: 277–283. 17611683

[41] Shah AN, Summy JM, Zhang J, Park SI, Parikh NU, et al (2007) Development and characterization of gemcitabine-resistant pancreatic tumor cells. Ann Surg Oncol 14: 3629–3637. 17909916 10.1245/s10434-007-9583-5

[42] Pirozzi G, Tirino V, Camerlingo R, Franco R, La Rocca A, et al (2011) Epithelial to mesenchymal transition by TGFbeta-1 induction increases stemness characteristics in primary non small cell lung cancer cell line. PLoS One 6: e21548. 21738704 10.1371/journal.pone.0021548 PMC3128060

[43] Argast GM, Krueger JS, Thomson S, Sujka-Kwok I, Carey K, et al (2011) Inducible expression of TGFbeta, snail and Zeb1 recapitulates EMT in vitro and in vivo in a NSCLC model. Clin Exp Metastasis 28: 593–614. 21643654 10.1007/s10585-011-9394-8

